# On the Narcotic Poisons, Particularly Opium, and Their Antidotes

**Published:** 1849-01

**Authors:** Francis Sibson


### MATERIA MEDICA AND THERAPEUTICS.

*On the Narcotic Poisons, particularly Opium, and their Antidotes.* By Francis Sibson.—It is my object in this, and the following papers on the same subject, to develope the results of an inquiry into the therapeutics of poisoning by narcotics, and more particularly by opium. That the most proper means of treating such cases are as yet either not agreed upon, or not generally known, is evidenced by the great variety of practice, and the frequent want of success in their treatment. The importance of the inquiry is shown by the very great number of persons that annually fall victims to poisoning by narcotics, especially by opium.

| | |
|---|---|
| In the years 1837–1838 the deaths by poisoning amounted to | 543 |
| Of these, the total number poisoned by opium, laudanum, morphia, and opiate cordials, were - - - | 198 |
| A number greater than that by arsenic - - - | 185 |
| Poisoned by other narcotics - - - - | 44 |
| viz., by Prussic acid - - - - - | 27 |
| Oil of bitter almonds - - - - - | 4 |
| Nux vomica - - - - - - | 3 |
| Strychnia - - - - - - | 2 |
| Belladonna - - - - - - | 2 |
| Carburetted hydrogen - - - - - | 2 |
| Hemlock, monkshood, wolfsbane, and gin, of each 1 - | 4 |

These returns (Medical Gazette, xxv. 284, and Taylor on Poisons, 186) show that the largest proportion of cases of poisoning in this country are by opium, exceeding even those by arsenic.

There can be no doubt, as Mr. Taylor remarks, that the number of deaths from poisons which annually occur in England and Wales are much greater than this table represents.

I may add, that this remark, applicable to all kinds of poisoning, is especially applicable to poisoning by opium, that drug being used so extensively by the ignorant, and acting so silently, and with so many of the appearances of natural death. The extent to which this is so, may be surmised from the fact, that of the 198 cases poisoned by opium, 106 were either from overdose or by mistake; 64 of the remainder being suicidal, and in 3 only was it "wilfully administered." So long as this is the only country in Europe where the sale of poisons is indiscriminate, we must expect that the number of persons poisoned by opium will be immeasurably greater in this country than in any other.

Since opium is the preponderating cause of death from narcotic poisoning in this country, I shall devote the chief portion of these papers to an inquiry into the action of opium as a poison, with the view of ascertaining the best means of averting its poisonous and fatal effects.

*On the local action of opium.*—Before endeavouring to ascertain the action of opium on the complicated human organism, I shall inquire into the evidences of its local action on separate portions of the animal organism.

During the last century, especially towards the latter part of it, many of the great physiologists of that day busied themselves with this very question, of the local action of opium and other agents. Amongst these were Whytt, Monro, Fontana, Alston, Valli, and Humboldt.

The numerous experiments and observations of Humboldt (Annals of Medicine, iv. 223-271) convinced him that opium, like other stimuli, exhausts only in consequence of excessive excitation. He exposed muscle shortly after its removal from the living frog to oxymuriatic acid: the effect was first to stimulate, and then to exhaust muscular contractility. This exhaustion may be removed, he found, by opium, which re-excites and then again exhausts contractility.

"This exhaustion may be removed, (he found) by oxymuriatic acid or oxide of arsenic, while opium also is capable of removing the inexcitability produced by them" p. 272. I ought to state that these are the words employed in the copious abstract of Humboldt's work in the Annals—the previous details being inferred, rather than actually extracted, from the statements in that abstract.

From the observations, then, of Humboldt, and of Michælis quoted by him, we may infer that the action of opium on the direct application of it either to nervous or muscular tissue, is first to augment, and then to exhaust their excitability.

These inferences are corroborated by the more recent experiments of Dr. Wilson Philip (on the Vital Functions, 133,) who observed, that when opium or tobacco are applied in very small quantity to a muscle, they tend to excite muscular action; in larger quantity they immediately destroy the muscular power. They produce these effects in the hollow muscles, as the heart and the intestines, chiefly when applied to their internal surfaces. They produce the same effect when applied locally to either the nervous or the sanguiferous systems.

In all these cases the stimulant effect of the opium is more considerable than that of the tobacco, and the sedative effect of the latter is more considerable than that of the former.

These observations of Dr. Wilson Philip differ in this respect from those of Humboldt, that while the latter noticed that opium first stimulates, and then exhausts excitability, the former noticed that the application of a small quantity of the poison immediately excites, whilst that of a large quantity immediately exhausts excitability. He does not state that the opium first excites and then exhausts.

Humboldt's and Wilson Philip's experiments taken together, illustrate the whole question, first in the application of a small quantity or dose—that is, in a therapeutical point of view—a small dose of opium being a true stimulant, as Sydenham said, almost the only true stimulant; while the application of a large quantity or dose exhibits the poisonous action.

Humboldt's experiments show that these two opposite actions are not really opposed to, but are dependent upon, and consecutive to, each other; and that, when the application of a large quantity of opium appeared to be immediately followed by exhaustion, that exhaustion was in reality preceded by the excitation, just as lightning in destroying vital contractility, primarily and violently excites it.

Valli (on Animal Electricity, 73) was much puzzled on finding that when he applied opium to the isolated tibial nerve of a frog, the excitability of the muscles of the leg was in some experiments destroyed, and in others increased. These experiments are explained by those of Humboldt and Wilson Philip. The cases in which excitability was increased were evidently in the earlier stages, and those in which it was destroyed in the latter stages of the action of the opium.

The great experimenters before alluded to, afford ample and interesting illustration of the local effect of opium in destroying excitability.

Whytt immersed the heart, still pulsating, of a frog in water, and another pulsating heart in a watery solution of opium. The heart in the watery solution ceased to pulsate before the heart in water.

In ten minutes both hearts were taken out: they were then motionless. That which had been in the watery solution of opiumcould not be stimulated to contract, and it never moved again; that which had been in water could be stimulated to contract—and in a few minutes it of itself resumed its pulsation. (Physical Essays, vol. ii.)

Monro poured ten drops of a watery solution of opium underneath the skin among the muscles of the left thigh. After ten minutes that leg seemed to be weaker, and in ten minutes more the muscles lost their power, and the toes had little sensibility: the animal seemed now to be a great deal stupified, and its heart gave now only 25 strokes in a minute. An hour and a half after the beginning of the experiment, the toes seemed to have quite lost their sensibility, and the muscles their motion; but the animal jumped by the help of the other hind extremity. Two days thereafter this leg had recovered its sense and motion, and the animal seemed quite well. (Physical Essays, xiv. 827.) Fontana says, (on Poisons, ii. 364,) "I plunged half the body of a leech into a watery solution of opium, and found in a little time that this part had lost all motion, whilst the other half continued in action. I looked upon it as something very extraordinary, that one half of the creature should become dead, whilst the other half continued in the state of not having undergone any change or suffered any injury."

The interesting experiments detailed above, prove that almost every and any organ of the body may be affected by the local action of opium; and that the organ affected by it has its excitability first increased, and then exhausted.

If the poison be applied to the voluntary muscles, or to the heart or intestines, the muscular contractility is first excited, and then exhausted. If it be applied to the individual nerves, or to the whole limb, sensation and motion are first excited, and then paralysed.

There is now no occasion to bring forward proof that the narcotic poisons as well as all other soluble substances, when received into the stomach or into the rectum, and in some animals, and with some agents when applied to the skin, pass into the circulation.

As the opium enters the blood when it acts upon the system, it necessarily follows that the opium admitted into the circulation is carried with the blood to every organ and portion of the frame. Every part of the organism is subjected to the immediate and characteristic influence of the poison; and as Whytt says, opium destroys by rendering the several organs insensible to the stimuli destined by nature to excite them.

As the opium when admitted into the system is first diffused through and applied to the whole circulating apparatus, I shall first inquire into the effect of opium on the circulation in the capillaries, arteries, and veins.

*The effect of opium on the circulation.*—Dr. Allston, that he might observe the effect of opium on the circulation in frogs, performed the following beautiful experiments, which he thus details:—

"(α) In the physic garden at Holyrood House, I one evening put a strong big paddock into a pot of water, wherein a small quantity of opium was dissolved; it soon appeared uneasy, by making strong efforts to get out of it, but in a short time it flagged or grew dull, making very little motion, and next morning it was dead, and much swelled." This experiment proves that opium can enter the circulation through the integuments, and so destroy life. This had not hitherto, I believe, been demonstrated.

"(β) Assisted by Mr. Robert Fullerton, a curious gentleman, and very dexterous in microscopical observation, (in August, 1733,) I conveyed through a small glass tube, a few drops of a solution of opium in water into a frog's stomach, and putting the animal into a glass cylinder, adapted it so to a good microscope that we had a distinct view of a part of the membrane betwixt the toes of its hinder foot, where the circulation of blood may easily be seen. My design was, since I found opium killed frogs, to observe if there was any visible change made by it in the blood itself, or in its motion; neither of us could, indeed, see any alteration in the blood as to its consistence, colour of the serum, magnitude, figure or colour of the red globules; but we very distinctly saw a surprising diminution of the blood's velocity; for it did not move half so swiftly as it used to do in these creatures. We alternately looked at it again and again, and in less than half an hour saw the velocity of the blood gradually increase, the uneasy frog recover its wonted vigour, and the blood its common celerity; upou which we took out the paddock, put it in a bason of clean water, and allowed it half an hour to refresh itself—then gave it another dose of opium—fixed it to the microscope with all expedition—and viewed it as before. The blood then moved, yet slower than it did the first time, and, its velocity gradually decreasing, at length it stagnated, first in the smaller, then in the larger vessels, and in about a quarter of an hour the animal expired.

"One thing was very observable all along—viz. that notwithstanding the diminished velocity of the blood, there was no sensible diminution of the frequency of the pulse: yea, when there was no circulation or progressive motion of the blood in this part, the pulse was visible by an undulatory motion—that is, the blood returned as far back at every diastole of the heart as it was protruded by the preceding systole: this continued too, till the frog was quite dead, or at least appeared to be so. When we had lost all hope of its recovery, I opened it, and found nothing in its stomach but a clear mucus like a jelly, a little coloured with the opium of which it was full: everything else seemed perfectly natural. This experiment we frequently repeated, and it had always the same appearance and event. The recovery, however, of one of the frogs which for a considerable time seemed to be dead, is not to be omitted. My friend and I one evening killed, as before, a couple of frogs with opium: one of them, which was the strongest, I laid half in water on a tile in the bottom of the water-pot, that if it recovered it might sit either wet or dry as it liked best; the other I left on the earth, dry, under a hedge. Next morning when I returned to the garden, I found the one under the hedge dead, as I left it; but the other in the water-pot was alive, and appeared to be in perfect health." (Medical Essays, v. 130.)

These two very interesting experiments, which for their real illustrative value, in elucidating the action of opium on the system, as well as for their historical interest, are well worth repeating here, I combined into one experiment, which I devised in the following manner. The experiment is easily performed:—

*Experiment.*—I attached a frog to Mr. Goadby's frog-holder, so that either web could be placed under the microscope. I plunged the left leg into a test-tube containing a watery solution of opium—the right leg into one containing water. The tubes were so arranged, that they could be withdrawn, and either webb be placed under the microscope without disturbing the frog.

Before the immersion of the left leg into the solution of opium, the circulation was very rapid: the corpuscles in the arteries shot past so rapidly that they could scarcely be distinguished. Those in the veins and the large capillaries moved rapidly, while those in the small capillaries moved slowly.

After the left leg had been immersed in the solution of opium for ten minutes, the motion of the blood in the smaller capillaries of that leg was quickened, and the blood circulated through many capillaries previously devoid of corpuscles. The movement of the blood in the artery and vein was less rapid. The circulation in the right leg was not altered.

After a further immersion of ten minutes the circulation in the left leg was further modified, that in the right leg being not perceptibly changed.

After the left leg had been replaced for half an hour, the frog was again observed. Whenever the skin was touched, either on the *left* or *right* leg, the frog cried out in a peculiar manner, the creature being universally convulsed. The skin was touched repeatedly, and in rapid succession, with the effect of producing convulsions, which became less and less strong each time they were excited. At length the convulsions could be no longer excited by touching the left leg, and after a time they ceased also to be excitable in the right leg. After a little rest the convulsions could again be excited.

The left leg was swollen, being evidently more vascular than the right.

The capillaries in the left leg were now much enlarged, several corpuscles moving slowly, side by side, through capillaries that were previously empty. The blood moved much more slowly both in the arteries and in the veins.

The circulation in the right leg was now very perceptibly modified, and, as nearly as it could be observed, to the same extent as that in the left leg was affected after being immersed in the solution of opium for ten minutes.

After a re-immersion for an hour and a half, quick feeble tetanic spasms of both limbs were excited by the slightest motion—by walking across the room, or touching the microscope, or by touching the skin of either leg. These convulsions ceased when the legs were touched alternately and in rapid succession; the left leg first lost its excitability, and then the right. The convulsive motions were the least in the left leg.

It was found during the last observation that the animal was quite unconscious, and had ceased to breathe.

The capillaries were now very much enlarged in the left leg, being greatly distended and almost blocked up with the accumulation of blood corpuscles, the motion of which was but just perceptible. The movement of the blood in the arteries and veins was exceedingly sluggish. The right leg was similarly affected, but the capillaries were not so much distended, and the circulation was not so slow, as they were in the left leg.

The circulation became progressively slower, and convulsions were no longer excitable. About four hours after the first immersion in opium the heart was exposed, pulsating slowly, emptying itself on each contraction, and receiving and sending out but little blood. After the heart was cut out, the movement of the blood in both webs continued, though it was very sluggish, and in the left leg was only observed in the large artery and vein.—*London Medical Gazette.*

